# An exploratory study of large language models in preoperative anesthesia assessment and planning for cardiac patients undergoing noncardiac surgery

**DOI:** 10.1186/s12871-026-04015-3

**Published:** 2026-06-13

**Authors:** Jianfan Ping, Yuanming Pan, Shuhan Gu, Hui Xu, Yongxing Yao, Diansan Su, Yueying Zheng

**Affiliations:** 1 Department of Anesthesiology, the First Affiliated Hospital, Zhejiang University School of Medicine, Hangzhou, Zhejiang 310003 P.R. China; 2 Zhejiang Key Laboratory of Clinical Evaluation Technology for Medical Device, The First Affiliated Hospital, Zhejiang University School of Medicine, Hangzhou, Zhejiang 310003 P.R. China

**Keywords:** Anesthesia assessment, Anesthesia plan, Large language models, Cardiac patients, Noncardiac surgery

## Abstract

**Background:**

With the rapid advancement of large language models (LLMs) in clinical applications, the potential role of LLMs in perioperative anesthesia assessment has garnered increasing research interest. This study aimed to systematically compare the performance, specifically accuracy and inter-model consistency of the three LLMs—ChatGPT, DeepSeek, and Grok—in performing preoperative anesthesia assessments and formulating anesthesia plans for cardiac patients undergoing noncardiac surgery, and to evaluate their agreement with an expert consensus panel.

**Methods:**

Forty-one consecutive patients with a history of severe cardiac disease, presented as standardized clinical vignettes derived from real medical records, were evaluated by the three LLMs, ChatGPT, DeepSeek, and Grok. Their outputs were compared against a structured reference standard derived from an expert consensus panel of five senior anesthesiologists. Agreement and accuracy were analyzed using Krippendorff’s α and Cohen’s κ coefficients, supplemented by qualitative thematic analysis.

**Results:**

DeepSeek achieved the highest accuracy in ASA classification (73.2%), followed by Grok (70.7%) and ChatGPT (58.5%). For the NYHA functional classification and RCRI scoring, Grok attained the highest accuracy in both tasks (75.6% for each), whereas ChatGPT exhibited the lowest accuracy (46.3%). In pulmonary risk assessment, Grok (80.5%) and DeepSeek (78.0%) outperformed ChatGPT (51.2%). Inter-model and model–expert agreement varied across the different assessment tasks, ranging from slight to substantial. All models exhibited a strong preference for recommending general anesthesia (≥ 85%) and consistently overemphasized invasive blood pressure (IBP) and central venous pressure (CVP) monitoring, while none mentioned bispectral index (BIS) monitoring.

**Conclusions:**

This exploratory study reveals that current LLMs exhibit suboptimal concordance with expert consensus in preoperative planning for complex cardiac cases. Although not ready for direct clinical application, their structured, rule-informed reasoning framework may serve as a supplementary checklist or second-opinion generator under expert supervision. Future multicenter validation studies incorporating diverse, high-quality datasets and standardized clinical endpoints are warranted to refine model training, improve generalizability, and substantiate clinical utility.

## Introduction

 In recent years, large language models (LLMs) have emerged as powerful tools transforming the medical field, with ChatGPT, DeepSeek, and Grok standing out as representative models with remarkable potential in clinical application. Developed by OpenAI, ChatGPT is widely recognized for its robust natural language understanding and generation functions, enabling it to assist in processing and analyzing complex anesthesia-related clinical text data [1, 2]. DeepSeek, an LLM independently developed in China, boasts the advantages of low training cost and high adaptability to medical scenarios, showing promising performance in tasks such as clinical data sorting and preliminary assessment [3, 4]. Grok, developed by xAI, excels in multimodal reasoning and handling complex clinical contexts, making it particularly suited to the dynamic and diverse demands of preoperative anesthesia assessment. Collectively, these LLMs show considerable promise in optimizing medical workflows, enhancing assessment efficiency, and supporting clinical decision-making, thereby laying a solid foundation for their application in specialized anesthesia practice.

Despite the rapid advancement and expanding applications of LLMs in healthcare, existing studies on LLMs’ use in preoperative anesthesia assessment and plan formulation remain limited in scope and depth. Most relevant research focused on the use of a single LLM (e.g., ChatGPT) in general anesthesia assessment scenarios [5, 6], or on routine preoperative evaluations for day surgery patients without severe comorbidities [7]. A growing body of research acknowledged the utility of artificial intelligence (AI) in the comprehensive preoperative assessment of high-risk patient populations—such as the geriatric and obstetric populations—yet a paucity of studies investigated the role of AI in preoperative evaluation for the broader cohort of patients with cardiovascular disease undergoing noncardiac surgery [8, 9]. Preoperative anesthesia assessment for this group is far more challenging: the interplay between cardiac comorbidities and surgical stress demands a more precise, comprehensive evaluation. Thus, there is an urgent need for targeted research to explore the applicability and effectiveness of LLMs in this specific clinical scenario.

Cardiac patients undergoing noncardiac surgery represent a clinically high-risk cohort with distinct characteristics: they often have complex cardiac comorbidities (such as coronary heart disease, heart failure, arrhythmia) and diminished physiological reserve capacity, making them highly susceptible to major adverse cardiac events during the perioperative period. Preoperative anesthesia assessment is a critical link in ensuring the safety of such patients, as it directly determines the formulation of individualized anesthesia plan and the reduction of perioperative risk. Although authoritative guidelines for the assessment and management of this patient population were established years ago [10, 11], current clinical preoperative anesthesia assessment still relies heavily on anesthesiologists’ personal experience and subjective judgment. This can lead to inconsistent assessment results, missed detection of potential risks, and challenges in achieving standardized, precise evaluation, highlighting the necessity of integrating more scientific, intelligent auxiliary tools.

To address the aforementioned research gap and meet the clinical demand for precise anesthesia assessment of cardiac patients undergoing noncardiac surgery, this single-center, small-sample exploratory study aimed to compare the performance of three mainstream LLMs—ChatGPT, DeepSeek, and Grok—in preoperative anesthesia assessment and anesthesia plan formulation for this specialized, complex population. By evaluating the accuracy, consistency, and practicality of these LLMs in handling relevant clinical tasks, this study aimed to clarify their advantages and limitations, provide valuable insights for the rational application of LLMs in the field of preoperative anesthesia management for this patient group, and ultimately promote the optimization of clinical anesthesia decision-making.

## Methods

Ethical approval was obtained from the Ethics Committee of the First Affiliated Hospital, Zhejiang University School of Medicine (Approval No. ITT20250521B). Informed consent was waived because all patient data were deidentified before analysis.

### Design

**Fig. 1:**
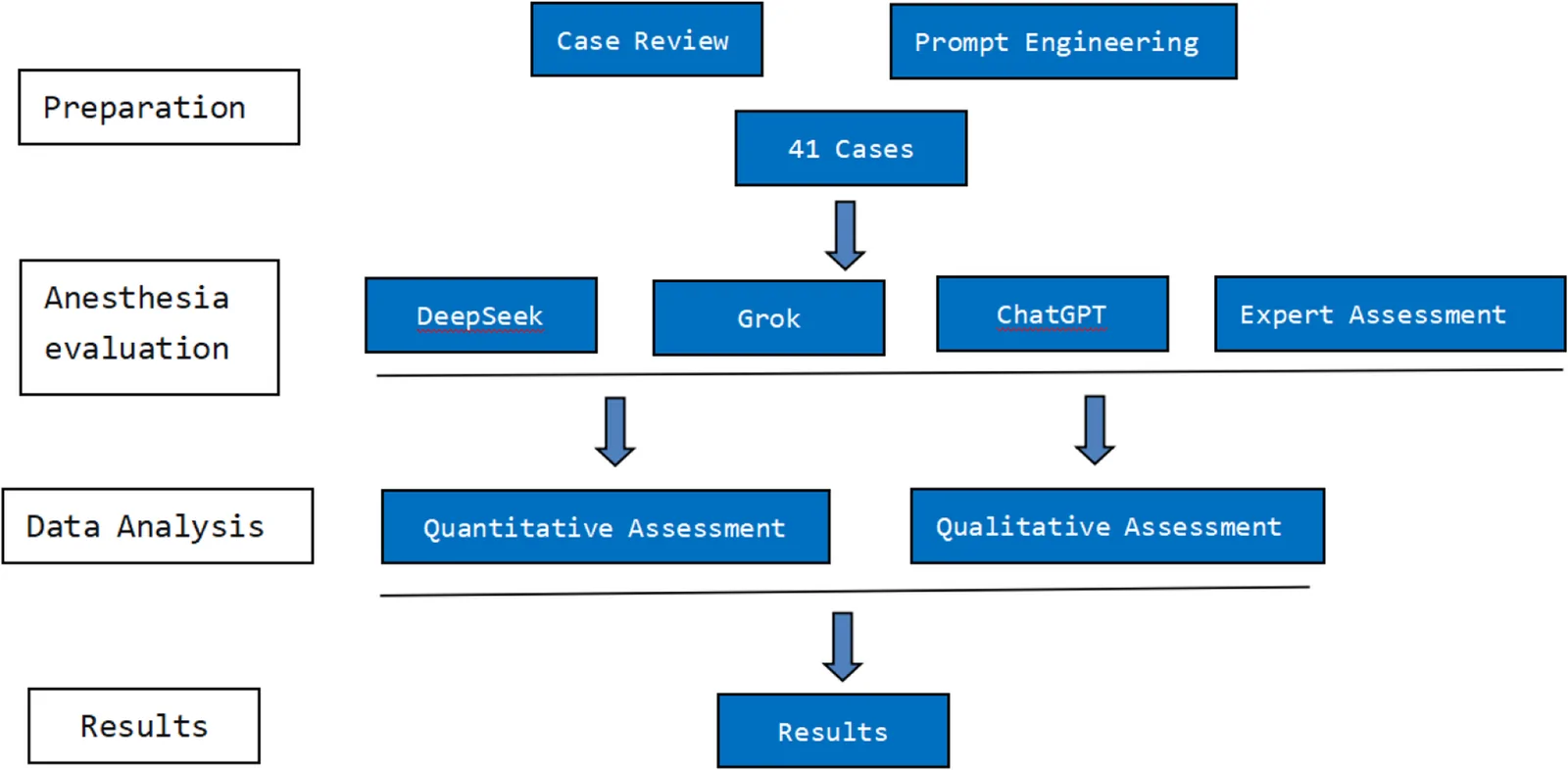
Flowchart of the study design. The study began with the preparation phase, which involved 41 clinical vignettes of real-world cases and prompt engineering. Forty-one cases were then randomly selected and presented to DeepSeek, ChatGPT, Grok and senior anesthesiologists to generate anesthesia assessment and anesthesia plans. Then the results from the AI models and the final expert were analyzed by qualitative and quantitative evaluation methods. The results from all assessments were then converged in the results section

This study adopted a comparative design to evaluate the accuracy and consistency of three large language models (LLMs), namely, ChatGPT (OpenAI, 2025), DeepSeek (DeepSeek, 2025), and Grok (xAI, 2025), in preoperative anesthesia assessment and the formulation of anesthesia plan for cardiac patients undergoing noncardiac surgery. We compared the preoperative anesthesia assessment results and anesthesia plans of 41 cases generated by these three LLMs with those produced by an expert panel comprising five senior anesthesiologists. A research flowchart was shown in Fig. 1. To prevent the AI from learning from our previous inputs and generated outputs during the study, and to eliminate model bias, all prompts provided to the three LLMs were given with the “chat history and training” settings disabled.

### Case selection

We selected 41 cardiac patients undergoing noncardiac surgery. In this study, “severe cardiac disease” was defined based on the 2022 ESC Guidelines and the 2024 AHA/ACC Guidelines [10, 11], including: (1) severe coronary artery disease (acute coronary syndrome, left main stenosis, or multivessel disease with symptoms/ischemia) or prior coronary stent implantation; (2) severe valvular heart disease meeting guideline indications for intervention (e.g., severe aortic/mitral stenosis or regurgitation with symptoms or left ventricular dysfunction); (3) decompensated heart failure; (4) complex congenital heart disease requiring intervention (e.g., cyanotic heart disease, significant shunt lesions with pulmonary hypertension, Fontan palliation, or Eisenmenger syndrome); (5) severe arrhythmias requiring treatment; and (6) severe pulmonary hypertension. In addition, patients with other high-risk conditions, such as recent stroke or frailty syndrome, as well as those with complex cardiac conditions who did not strictly meet these criteria but were deemed high-risk after formal multidisciplinary team (MDT) discussion, were also included to reflect real-world clinical decision-making. To avoid bias, the senior anesthesiologists forming the expert panel had no prior involvement in the care of these selected cases.

To generate the standardized input for the LLMs, a structured multi-step process was employed. First, two senior anesthesiologists independently extracted core preoperative information from the complete electronic medical records into a structured template. The extracted data included: (1) demographics (age, sex, BMI; with age ≥ 90 recorded as ‘≥90’ for de-identification), (2) cardiac history (diagnosis, time of diagnosis, and treatment details), (3) major comorbidities (e.g., hypertension, diabetes), (4) key preoperative test results (electrocardiography, echocardiography, chest CT when available, renal/hepatic function, coagulation profile), and (5) surgical information. Next, a third anesthesiologist synthesized these extracted data into a concise, narrative-style clinical vignette. The language was simplified to be conversational, simulating a real-world case presentation scenario between clinicians, while all personal identifiers (e.g., name, ID number) were removed. Finally, a fourth senior anesthesiologist reviewed the drafted vignettes against the original medical records to ensure completeness, accuracy, and fidelity, thereby minimizing potential bias or omission introduced during the summarization process. These finalized, de-identified vignettes served as the direct input for both the LLM prompts and the independent expert assessments.

### Prompts

**Fig. 2:**
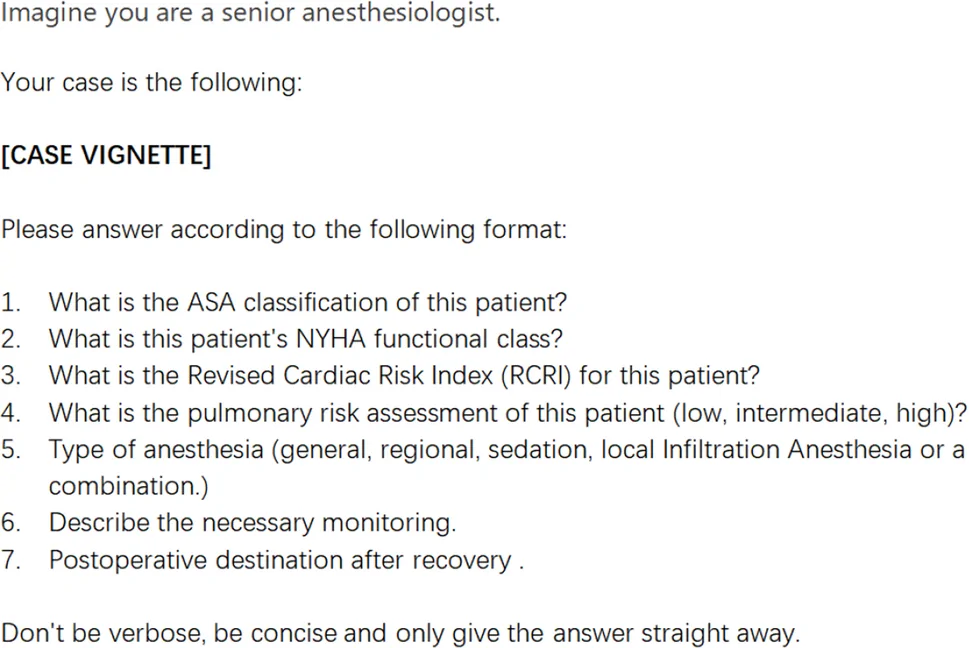
The structured prompt template provided to the large language models

We designed a fixed prompt instructing the three LLMs to perform preoperative anesthesia assessments and develop anesthesia plans for each case (Fig. 2). The anesthesia assessment included the American Society of Anesthesiologists (ASA) classification, New York Heart Association (NYHA) functional classification, Revised Cardiac Risk Index (RCRI), and pulmonary risk evaluation. The anesthesia plan covered the type of anesthesia (general anesthesia, regional anesthesia [including neuraxial or peripheral nerve blocks], monitored anesthesia care (MAC), or their combined regimens). Intraoperative monitoring included ASA-standard monitoring, invasive blood pressure (IBP), central venous pressure (CVP), and other grading schemes. The postoperative destination of the patient (general ward or intensive care unit [ICU]) was also part of the anesthesia plan. All prompts used a fixed structured template; chat history and fine-tuning were disabled. The structured prompt was developed through an iterative, collaborative process involving two additional senior anesthesiologists. To ensure clarity and effectiveness, the prompt underwent three rounds of pretesting with five non-study patient cases. This process confirmed that the prompt was consistently understood by all three LLMs and reliably elicited the structured output format required for the assessment.

### Analysis methods

This study utilized a mixed-methods approach combining quantitative and qualitative analyses. The reference standard was established by a panel of five senior anesthesiologists (each with > 15 years of experience) through a structured three-step process. Each expert independently assessed all 41 standardized clinical vignettes. Inter-rater reliability among the five experts was assessed using Krippendorff’s α. Discrepancies were subsequently resolved through a focused discussion based on the latest clinical guidelines, and the majority opinion was recorded as the “expert consensus result.”

### Quantitative analysis

For quantitative analysis, we compared LLM outputs with the expert consensus. Krippendorff’s α was used for both inter-expert (pre-consensus) and inter-model agreement. Cohen’s κ was used to measure model-expert agreement for each evaluation task.

### Qualitative and directed content analysis

To enable a structured, evidence-informed comparison of specific recommendations, we conducted a directed content analysis focused on intraoperative monitoring plans and postoperative destination. First, based on established clinical guidelines and expert consensus, we predefined two analysis dimensions: Monitoring Items (e.g., IBP, CVP, transesophageal echocardiography [TEE], BIS) and Postoperative Destination (ICU or general ward). Two independent researchers then systematically coded all LLM outputs, explicitly identifying whether each predefined item was recommended, omitted, or conditionally recommended. Inter-rater coding discrepancies were resolved through discussion to reach full consensus. Final consensus codes were used to calculate the recommendation frequency for each item by each LLM, enabling a direct quantitative comparison with the expert consensus frequencies, as reported in the Results section.

To externally validate the expert consensus, we compared its recommendations regarding postoperative destination with the actual clinical decisions documented in patients’ medical records, excluding cases in which surgery was ultimately not performed.

### Data analysis

This study presented both qualitative and quantitative results. For the qualitative results, descriptive analysis was used, and the final evaluator conducted a comprehensive thematic analysis of the collected qualitative data to identify commonalities and differences in the outputs of the three LLMs, as well as discrepancies compared to the evaluations of senior anesthesiologists. For the quantitative results, Krippendorff’s α was used to calculate the consistency in risk assessments. Statistical analyses were performed using R software (version 4.3.2, R Foundation for Statistical Computing, Vienna, Austria). The strength of agreement for Cohen’s κ was interpreted using the guidelines proposed by Landis and Koch: 0.81–1.00 almost perfect; 0.61–0.80 substantial; 0.41–0.60 moderate; 0.21–0.40 fair; ≤0.20 slight [12]. Krippendorff’s α was interpreted using similar thresholds.

### AI models

The ChatGPT model version is ChatGPT-5 mini (OpenAI).

The DeepSeek model version is DeepSeek V3 (DeepSeek).

The Grok model version is Grok-3 (xAI).

All models were accessed via their official interfaces in October 2025. To leverage their advanced reasoning functions for clinical scenario analysis, we enabled each model’s built-in chain-of-thought mode (referred to as “advanced reasoning” in ChatGPT, ‘deep thinking’ in DeepSeek, and a similar feature in Grok) by selecting the corresponding option in the user interface before inputting the prompts. All other parameters were kept at their default settings, and models were used in their publicly available, non-fine-tuned form.

## Results

### Expert inter-rater reliability prior to consensus

Prior to the consensus discussion, inter-rater reliability among the five independent experts was assessed using Krippendorff’s α. The resulting coefficients were: ASA classification (α = 0.806), NYHA functional classification (α = 0.946), pulmonary risk assessment (α = 0.922), RCRI scoring (α = 0.877), and anesthesia method selection (α = 0.875) (Table 1).

**Table 1:** Expert inter-rater reliability prior to consensus discussion

Assessment Metric	Inter-Expert Krippendorff’s α
ASA Classification	0.806
NYHA Functional Classification	0.946
Pulmonary Risk Assessment	0.922
RCRI Scoring	0.877
Anesthesia Method Selection	0.875

*Abbreviations*: *ASA* American Society of Anesthesiologists, *NYHA* New York Heart Association, *RCRI* Revised Cardiac Risk Index

### Risk assessment

The accuracy of ASA classification against the expert consensus was highest in DeepSeek (73.2%, 30 correctly classified cases), followed by Grok (70.7%, 29 cases) and ChatGPT (58.5%, 24 cases). The consistency among the three LLMs in ASA scoring was moderate (Krippendorff’s α = 0.496). Furthermore, agreement with expert consensus in ASA scoring was moderate for DeepSeek (κ = 0.52), and fair for Grok (κ = 0.388) and ChatGPT (κ = 0.316).

The accuracy of NYHA functional classification against the expert consensus was highest in Grok (75.6%, 31 correctly classified cases), followed by DeepSeek (73.2%, 30 cases) and ChatGPT (46.3%, 19 cases). Inter-model consistency for the NYHA classification was substantial (Krippendorff‘s α = 0.682). Agreement with expert consensus, measured by Cohen‘s κ, was substantial for Grok (κ = 0.739) and DeepSeek (κ = 0.700), but only fair for ChatGPT (κ = 0.360).

The accuracy of the Revised Cardiac Risk Index (RCRI) calculation against the expert consensus was highest for Grok (75.6%, 31 correctly classified cases), followed by DeepSeek (70.7%, 29 cases) and ChatGPT (46.3%, 19 cases). Inter-model consistency in RCRI scoring was moderate (Krippendorff‘s α = 0.591). Agreement with expert consensus was moderate for Grok (κ = 0.644) and DeepSeek (κ = 0.558), and slight for ChatGPT (κ = 0.280).

In the assessment of pulmonary risk, accuracy rates varied across models: DeepSeek (78.0%) and Grok (80.5%) showed similar and higher agreement with experts, while ChatGPT‘s accuracy was lower (51.2%). The consistency among the three LLMs was substantial (Krippendorff’s α = 0.735). However, the consistency between each model and the clinical experts in pulmonary function assessment was substantial for DeepSeek (κ = 0.740) and Grok (κ = 0.741), and moderate for ChatGPT (κ = 0.438). The overall performance summary is presented in Table 2.

**Table 2:** Summary of performance on the risk assessment task

Assessment Metric	Model	Cases in Agreement, *n* (%)	95% CI for Accuracy	Cohen‘s κ(95% CI)
ASA	DeepSeek	30 (73.2%)	58.0% – 84.3%	0.520(0.308–0.738)
	Grok	29 (70.7%)	55.5% – 82.4%	0.398(0.137–0.639)
	ChatGPT	24 (58.5%)	43.3% – 72.2%	0.316(0.086–0.546)
NYHA	DeepSeek	30 (73.2%)	58.0% – 84.3%	0.700(0.539–0.862)
	Grok	31 (75.6%)	60.7% – 86.2%	0.739(0.593–0.886)
	ChatGPT	19 (46.3%)	32.1% – 61.3%	0.360(0.171–0.550)
RCRI Score	DeepSeek	29 (70.7%)	55.5% – 82.4%	0.558(0.358–0.758)
	Grok	31 (75.6%)	60.7% – 86.2%	0.644(0.450–0.838)
	ChatGPT	19 (46.3%)	32.1% – 61.3%	0.280(0.100–0.460)
Pulmonary Risk	DeepSeek	32 (78.0%)	63.3% – 88.0%	0.740(0.583–0.897)
	Grok	33 (80.5%)	66.0% – 89.8%	0.741(0.573–0.909)
	ChatGPT	21 (51.2%)	36.5% – 65.7%	0.438(0.282–0.594)

Data are presented as n (%) unless otherwise specified. Accuracy indicates the proportion of cases where the model’s assessment matched the expert consensus. Cohen’s κ was interpreted according to Landis and Koch (1977): 0.81–1.00, almost perfect; 0.61–0.80, substantial; 0.41–0.60, moderate; 0.21–0.40, fair; ≤0.20, slight. Cohen’s κ values are color-coded to indicate agreement strength (light green: ≤0.40; medium green: 0.41–0.60; dark green: ≥0.61)

*Abbreviations:* *ASA* American Society of Anesthesiologists, *CI* Confidence interval, *NYHA* New York Heart Association, *RCRI* Revised Cardiac Risk Index

### Anesthesia method

In the choice of anesthesia methods, DeepSeek achieved the highest accuracy (87.8%), which agreed with expert consensus on 36 cases; Grok and ChatGPT followed (85.4%), both of which agreed with expert consensus on 35 cases. The inter-model consistency was substantial (Krippendorff’s α = 0.754). However, the consistency between DeepSeek, Grok, and ChatGPT and the clinical experts in the choice of anesthesia method was fair for DeepSeek (κ = 0.285), and moderate for Grok (κ = 0.452) and ChatGPT (κ = 0.532). The results for anesthesia method selection are presented in Table 3.

**Table 3:** Summary of performance on the anesthesia method

Model	Cases in Agreement, *n* (%)	95% CI for Accuracy	Cohen‘s κ(95% CI)
DeepSeek	36 (87.8**%**)	74.4% – 94.7%	0.285(−0.054–0.624)
Grok	35 (85.4**%**)	71.6% – 93.1%	0.452(0.101–0.803)
ChatGPT	35 (85.4**%**)	71.6% – 93.1%	0.532(0.203–0.861)

Data are presented as n (%) for agreement counts and as percentages with 95% confidence intervals where indicated. Accuracy indicates the proportion of cases where the model’s anesthesia method recommendation matched the expert consensus. Cohen’s κ was interpreted according to Landis and Koch (1977) (see Table 1 note for details)

*CI C*onfidence interval

### Perioperative monitoring

As part of the qualitative thematic analysis, the monitoring recommendations of the three LLMs were coded and analyzed, and the results were compared with the expert consensus. All three models were consistent with the expert consensus in advocating standard ASA monitoring for every patient. This study identified notable variations in intraoperative monitoring strategies among the three LLMs, with a general tendency toward overuse of invasive monitoring. The DeepSeek model inclined toward comprehensive monitoring protocols, frequently supplementing standard ASA monitoring with invasive arterial pressure monitoring (39/41, 95.1%), CVP monitoring (26/41, 63.4%), and, in some cases, TEE (11/41, 26.8%). The Grok model produced recommendations similar to those of DeepSeek, predominantly based on standard ASA monitoring with added invasive arterial pressure monitoring (34/41, 82.9%), occasional inclusion of CVP monitoring (30/41, 73.2%), and a very low rate of TEE recommendation (1/41, 2.4%). In contrast, the ChatGPT model adhered to a highly standardized approach, universally recommending standard ASA monitoring combined with invasive arterial pressure monitoring (41/41, 100%), CVP monitoring (35/41, 85.4%), and TEE (23/41, 56.1%).

### Postoperative destination

As part of the qualitative thematic analysis, the postoperative destination recommendations of the three LLMs were coded and analyzed, and the results were compared with the expert consensus. Regarding postoperative destination recommendations, the expert consensus advised ICU admission for 9 of 41 patients (22.0%) and routine ward care for 32 patients (78.0%). In contrast, DeepSeek, Grok, and ChatGPT recommended ICU admission for 37 patients (90.2%), 30 (73.2%), and 35 (85.3%), respectively.

### Validation of expert consensus against actual clinical decisions

To assess the external validity of the expert consensus as a reference standard, we compared its postoperative destination recommendations with the actual clinical decisions documented in medical records. After excluding 7 cases in which surgery was not ultimately performed, 34 cases were available for this comparative analysis.

The expert consensus demonstrated a high overall agreement rate of 91.2% (31/34 cases) with actual clinical practice. Specifically, in 30 cases (88.2%), the expert panel and actual practice recommended ward care, while in 1 case (2.9%), recommended ICU admission. Discrepancies occurred in only 3 cases (8.8%): the expert panel recommended ward care while the actual decision was ICU admission in 2 cases, and vice versa in 1 case. The expert panel recommended ICU admission for only 5.9% (2/34) of cases, which was comparable to the actual ICU admission rate of 8.8% (3/34).

## Discussion

Despite the proliferation of LLM evaluations in anesthesia, comparative studies focusing on patients with severe cardiac disease undergoing noncardiac surgery remain limited. This study provided a head-to-head comparison of ChatGPT, DeepSeek, and Grok in this clinically important population, extending the literature by evaluating model performance across structured risk scoring and individualized planning. Our findings highlighted both the potential and important limitations of current LLMs in complex perioperative decision-making.

Overall, the three LLMs showed variable performance in preoperative risk assessment. While DeepSeek and Grok achieved moderate accuracy (> 70%) in structured scoring tasks, including ASA and NYHA classifications, all models performed poorly in individualized anesthesia planning, suggesting that current LLMs lack the contextual reasoning required for safe clinical deployment in high-risk perioperative settings. Consistent with Jarrassier et al.’s findings [13] (where no LLM was reliable for complex/high-risk patients despite ChatGPT’s superior guideline adherence), our study advances the field by analyzing real cases of cardiac patients undergoing noncardiac surgery and evaluating the understudied DeepSeek and Grok. Collectively, both studies demonstrate that current LLMs are unfit for autonomous personalized perioperative planning for complex patients.

For the ASA classification, the accuracy ranged from 58.5% (ChatGPT) to 73.17% (DeepSeek), consistent with a previous finding [6]. Although ASA grading is widely considered straightforward, misclassification is common among ASA III-IV patients due to complex comorbidities [14]. LLMs frequently encounter logical conflicts when integrating multidimensional clinical information, contributing to reduced accuracy in high-risk subgroups. A prospective multicenter study using optimized ChatGPT-4 reported almost perfect agreement with anesthesiologists. However, performance declined markedly in complex cases such as ASA IV patients. Similarly, an exploratory study in pediatric populations demonstrated only moderate agreement [15]. In contrast, our study observed only fair agreement (κ = 0.316) using a general-purpose ChatGPT in high-risk cardiac patients. Variability across studies may reflect differences in model version, input structure, and study design, emphasizing the need for model optimization and standardized evaluation in high-risk populations.

For NYHA classification, which relies on subjective functional status, substantial variability among experts and LLMs is expected. In contrast, RCRI is an objective scoring system, yet ChatGPT achieved only 46.3% accuracy, substantially lower than DeepSeek (70.7%) and Grok (75.6%). ChatGPT frequently over-interprets or conflates general cardiovascular risk factors such as diabetes or hypertension that are not components of the RCRI, indicating difficulty in strictly adhering to defined clinical scoring criteria. All three LLMs mechanically interpreted a reduced left ventricular ejection fraction (31%) as indicative of congestive heart failure for RCRI purposes without considering functional status or formal criteria. These discrepancies likely reflect differences in training data composition, guideline coverage, and algorithmic logic, the precise contributions of which remain speculative without full model transparency.

All LLMs exhibited excessive reliance on general anesthesia, with regional or combined anesthesia recommended in fewer than 5% of cases, consistent with prior reports [5]. ChatGPT showed repetitive recommendations for general anesthesia and lacked multimodal analgesia strategies, possibly due to limited regional anesthesia content in training data. All models over-recommend invasive monitoring (e.g., IBP, CVP) without adequate individualized justification. DeepSeek recommended TEE even in low-risk laparoscopic surgery, while experts deemed standard ASA monitoring sufficient. Notably, none of the models recommended Bispectral Index (BIS) monitoring, which may reflect limited guideline coverage in training data or strict interpretation of ‘necessary monitoring’ rather than a pure knowledge deficit. This underscores the importance of prompt engineering and individualized perioperative management. LLM-generated plans should be viewed as adjunctive guidance only.

Regarding anesthesia method selection, although raw accuracy was high (85.4–87.8%), Cohen’s κ values were only fair to moderate (0.285–0.532). This discrepancy is largely due to severe class imbalance: general anesthesia was recommended by expert consensus in 85.4% (35/41) of cases. A model could thus achieve high accuracy by mostly recommending general anesthesia, but κ remains low because it penalizes poor performance on the minority class (regional or combined anesthesia). All three models indeed recommended general anesthesia in ≥ 85% of cases, confirming this bias.

Regarding postoperative destination, all three LLMs demonstrated strong conservative bias, with ICU admission rates ranging from 73.2% to 90.2%, markedly higher than the expert rate of 22%. This overtriage tendency aligns with studies reporting high sensitivity but low specificity for ICU prediction [16]. Notably, in the subset of cases where actual clinical decisions were documented (*n* = 34), both expert consensus (5.9%) and actual practice (8.8%) overwhelmingly preferred ward care. The lower actual ICU admission rate (8.8% vs. the expert-recommended 22%) was largely due to uneventful intraoperative recovery and limited ICU bed availability, which led to an attempt at extubation in the post-anesthesia care unit. Although Grok showed slightly better discrimination in selected low-risk cases with correctly recommended ward discharge in Patient 8, all models favored overly conservative disposition. Such decisions remain institution-dependent based on resource availability and local clinical practices.

Despite strong performance in fundamental medical knowledge and standardized tests [14, 17], this study confirms that LLMs still exhibit significant limitations in complex, individualized clinical reasoning. These findings are consistent with a previous study that revealed suboptimal accuracy of LLMs in specialized cardiothoracic anesthesiology knowledge assessments [18].

Several limitations warrant consideration. First, this was a single-center, small-sample study (*n* = 41) comprising mainly elective laparoscopic surgeries without emergency scenarios; therefore, any comparative conclusions drawn from this study should be interpreted with caution, as observed differences may be due to chance or local practice patterns. Second, the reference standard came from a single institution, introducing potential circularity and reflecting local rather than universal practice; post-hoc validation (*n* = 34) was insufficient to resolve this. Third, the study focused only on medically complex cardiac patients undergoing noncardiac surgery and used only free-access LLMs, all of which limit generalizability. Accordingly, our findings serve primarily to generate hypotheses. Fourth, cross-language and cross-cultural variations in guidelines were not addressed, and standardized clinical vignettes may have omitted subtle details present in full electronic records. Fifth, no formal pairwise comparisons were performed (observed differences among models may be due to chance), chain-of-thought prompting was enabled (its effect relative to standard prompting unknown), and perioperative guidelines were not provided nor retrieval-augmented prompting used (which may have skewed outputs toward institution-dependent patterns). Future research should aim to minimize unnecessary simplification, better preserve critical clinical details, adopt retrieval-augmented prompting for fairer assessment, explore alternative input methods such as structured data templates or secure, de-identified raw data interfaces, and use multicenter, guideline-based validation.

Owing to the training data limitations and AI hallucination, LLMs may generate inaccurate or misleading recommendations in clinical scenarios [19, 20]. Our findings show that current general-purpose LLMs are not ready for independent clinical use in high-risk perioperative scenarios. At this stage, their role is best characterized as a supplementary checklist or second-opinion generator under expert supervision, rather than as a tool for autonomous clinical decision-making. The future research should focus on specialized training with diverse, guideline-updated cases, multidisciplinary model refinement, and integration into structured clinical decision support systems rather than standalone application. Long-term real-world validation is essential to improve reliability and ensure safe clinical translation.

## Data Availability

All data supporting the findings of this study, namely the 41 de-identified clinical vignettes (standardized case summaries) used to prompt the large language models, are available within this article and its supplementary information files (Additional File 1: CASE.doc).
